# Analysis of bone ingrowth on a tantalum cup

**DOI:** 10.4103/0019-5413.39553

**Published:** 2008

**Authors:** F D'Angelo, L Murena, M Campagnolo, G Zatti, P Cherubino

**Affiliations:** Department of Orthopedics and Traumatology, University of Insubria, Varese, Italy

**Keywords:** Bone ingrowth, fixation, tantalum

## Abstract

**Background:**

Trabecular Metal (TM) is a new highly porous material made of tantalum (Zimmer, Warsaw, Indiana, USA). Its three-dimensional structure is composed of a series of interconnected dodecahedron pores that are on average 550 μm in diameter. This size is considered optimal for bone ingrowth and is similar to trabecular bone. The elastic modulus of TM (3 GPa) is more similar to that of cancellous (0,1-1,5 GPa) or cortical (112-18 GPa) bone and is significantly less similar to that of Titanium (110 GPa) and Co-Cr alloys (220 GPa). These features enable bone apposition and remodeling. The purpose of the present study was to evaluate the histology of the bone-implant interface in a human specimen.

**Materials and Methods:**

A highly porous tantalum cup (Zimmer, Warsaw, Indiana, USA) was removed for recurrent dislocations three years after implantation. In order to obtain a slice of the cup, two cuts were made on the centre using an Exakt cutting machine. Then the slice was embedded in a Technovit resin and a Hematoxylin-eosin stain was used to study the bone tissue. Bone ingrowth was calculated using a method based on simple calculations of planar geometry.

**Results:**

The histological evaluation of the periprosthetic tissues revealed a typical chronic inflammation with few particles of polyethylene that were birefringent using polarized light. The quantitative evaluation of bone ingrowth revealed that more than 95% of voids were filled with bone.

**Discussion:**

In the literature, a lot of studies focused on tantalum were carried on animal model. Up to now little information is available about the histology of the bone-tantalum interface in a human artificial joint. We had an opportunity to remove a well integrated cup hence this study. The histology confirmed the strong relationship between the structure of this material and bone. The morphometric analysis revealed a high percentage of bone ingrowth.

## INTRODUCTION

In the last 20 years a variety of porous surfaces and materials has been used to obtain fixation with bone ingrowth in total hip and knee prostheses. The most common include titanium and cobalt-chrome-alloy sintered beads, diffusion-bonded titanium, fiber metal and titanium plasma spray.

Based on animal models, clinical studies and evidence from retrieved implants, it is clear that porous surfaces support tissue ingrowth or ongrowth and are generally effective for supplementing the primary mechanic fixation by osteointegration.

A new porous biomaterial made of tantalum has recently been developed for potential application in reconstructive orthopedics and other surgical disciplines. This material is a carbon scaffold covered by pure tantalum. The main features are: a high porosity up to 80%, a lower elastic modulus (3 GPa) and a high frictional stability against bone. Trabecular metal has an unusually large and interconnecting porous surface which corresponds to between 75 and 80% of its total volume and an overall geometry, shape and size similar to cancellous bone. The high-volume porosity enables extensive tissue infiltration and strong attachment. The microtexture of trabecular metal is osteoconductive. The metal elasticity of trabecular bone is 3 GPa, which is between that of cancellous (0.1 GPa), subchondral (2 GPa) and cortical bone (15 GPa). Titanium alloys (110 GPa) and cobalt-chromium alloys (220 GPa) are much less elastic.^1^

Several studies have been published on this new material, taking into consideration animal models or clinical series.^1^–^5^ Bobyn studied the characteristics of bone ingrowth of tantalum in a canine model using first, cylinder implants^1^ and then a fully functional total hip arthroplasty model^2^ demonstrating high extent of bone filling. In a multicentric study Gruen *et al.*,^3^ evaluated a monoblock acetabular component reporting encouraging results despite the short follow-up (two to five years). Macheras *et al.*,^4^ undertook a radiological study to evaluate the osteoconductive properties of the tantalum bone interface and the possibility to fill an initial gap. Komarasamy *et al.*,^5^ evaluated 113 consecutive tantalum monoblock cups showing that in the short term the new uncemented tantalum/polyethylene composite acetabular component can yield a satisfactory clinical and radiological outcome and has a high patient satisfaction.

The use of tantalum is also reported in revision hip arthroplasty where the acetabular defects are treated by means of hemispherical cup and augment. Paprosky *et al.*,^6^ reported encouraging results in a series of 28 hips with Type IIIA acetabular defects, at three years of follow-up. Even during revision total knee replacement the porous tantalum cones have been used to treat large tibial bone defect. There was no sign of loosening or migration in a population of 15 knees at three years.^7^

The purpose of the present study was to evaluate the histology of the bone-implant interface in a human specimen.

## MATERIALS AND METHODS

A 64-year-old man was treated with a primary uncemented total hip arthroplasty using a trabecular monoblock acetabular component and a Versys ET stem (Zimmer^®^, Warsaw, Indiana, USA) at our department. The porous tantalum acetabular monoblock component used is hemi-ellipsoid-shaped; the diameter of its equator is 2 mm larger than its polar diameter. It is inserted using a press fit technique, taking particular care to prevent the interposition of soft tissue. The polyethylene liner is compression molded into the shell to a depth of 1 to 2 mm, leaving 2 to 3 mm of porous tantalum for tissue ingrowth. Three years later the acetabular component was revised for recurrent dislocations without signs of loosening.

**Figure 1:**
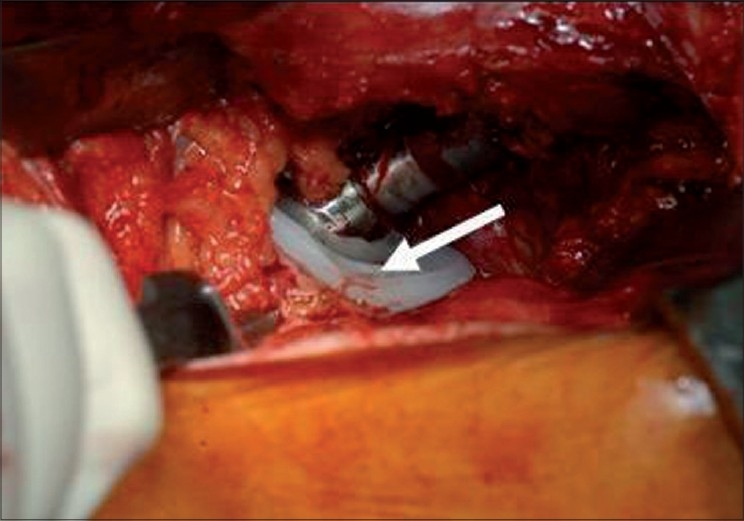
Intraoperative view: the zone of impingement between the elevated liner and the neck is clearly visible (solid arrow)

The revision was performed through a conventional postero-lateral approach. The inspection of the joint space revealed that the cause of the dislocations was an impingement between the liner and the neck of the stem [Figure 1]. The acetabular component was well fixed to the bone and was removed using a dedicated instrumentation (Explant Acetabular Cup Removal System, Zimmer^®^, Warsaw, Indiana, USA) which cut the bone around the prosthesis.

A sample of the neocapsule was harvested, fixed in formalin and prepared for histology in order to look for polyethylene debris. After the disydratation phase in a rising scale of alcohol, the samples were embedded in paraffin and stained with ematoxilin-eosin.

Following fixation in 10% buffered formalin solution, the cup removed was prepared for histological processing. The acetabulum was sectioned in a coronal plane using a diamond saw (Exact) to yield a single section of the cup. Then it was dehydrated and embedded in Technovit resin. After polimerization, it was grinded up to 100 µm, stained with ematoxilin-eosin and examined with light microscopy.

**Figure 2:**
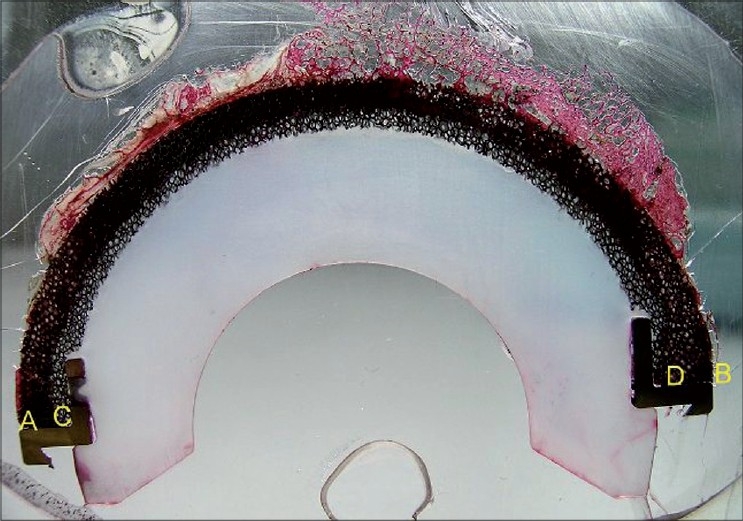
A view of the section of the cup embedded and already stained. The letters AB identify the diameter of the external hemicircle while CD the internal one

In order to obtain a quantitative evaluation of the bone ingrowth we used a personal geometrical method. We calculated the area of tantalum available for bone ingrowth identified by the difference of the areas of the two semicircles identified by the two diameters AD and BC [Figure 2]. Afterwards, we calculated the number of the non-bone areas identified as non-stained pores and their cumulative area was related to the total area in order to obtain the percentage of non-bone area.

## RESULTS

**Figure 3:**
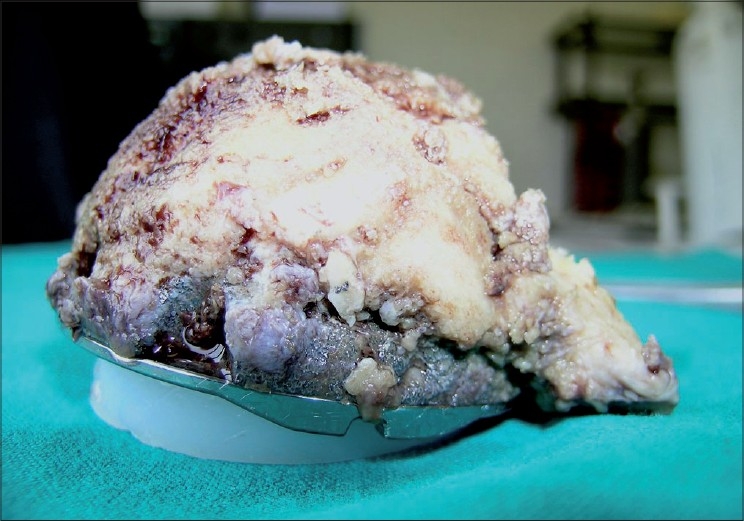
After the removal was completed, the cup was extensively covered by bone

At the time of revision the cup was extensively and firmly covered by bone [Figure 3].

**Figure 4:**
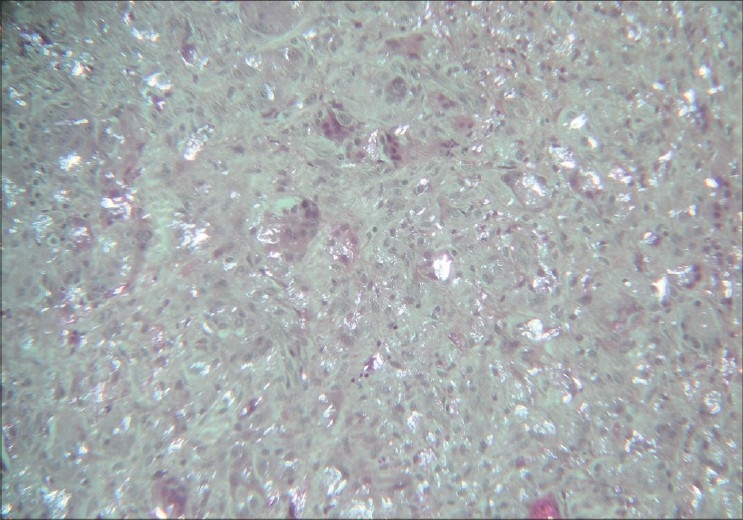
Polarized light microscopy revealed the presence of debris of polyethylene in the neocapsule (–25)

**Figure 5:**
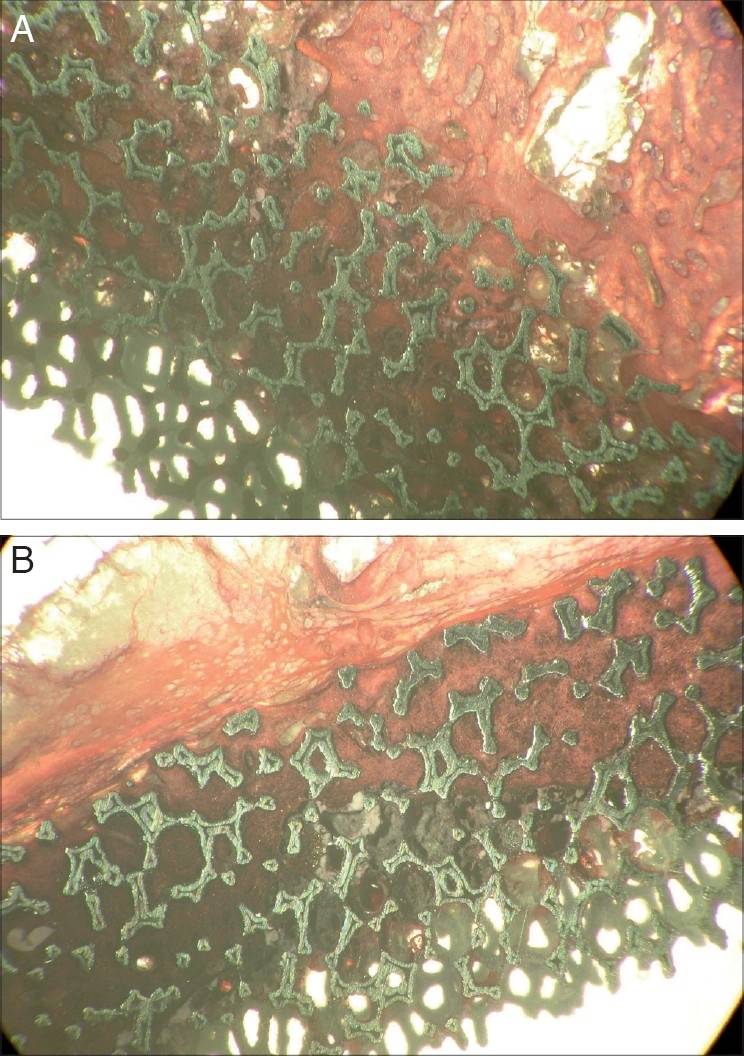
(A and B) Two different regions of the cup covered by bone

The polarized light microscopy revealed the presence of polyethylene at the site of the neocapsule with all the aspects of a chronic inflammatory response with multinucleate cells and cells of the macrophagic system [Figure 4]. The light microscopy revealed the presence of new bone within the pores of the material. The extent of ingrowth varied along the circumference, tending to be higher at the level of the dome region [Figures 5A, B]. The quantitative evaluation of the extent of bone ingrowth gave a value higher than 90%.

## DISCUSSION

The performance of a new material can be studied in various ways. Animal models offered the researcher the possibility of assessing its performance while *in vivo*. However, the different biomechanics of the animals could interfere with the ingrowth process or could influence different results.

Radiographic analysis allows orthopedists to asses the performance, but the two-dimensional nature of radiographs cannot provide any information of the nature of the interface tissue.

In our country, we have no possibilities to analyze components retrieved at autopsy, losing the opportunity to understand the histology of the interface of a well-fixed component. On the other hand, we have an extensive experience on the interface of loosened components. Even in these cases, the limit is that the material has failed and the researchers have little information about ingrowth.

The tantalum is a new porous material recently developed for application in reconstructive orthopedics. The greater porosity of this material (almost 85%), when compared with either fiber metal (45-50%) or sintered beaded coatings (30-35%), gave the possibility of larger extent of new bone formation within the pores. This could cause an increase of the interface mechanical strength.

Bobyn *et al.*,^1^ studied the characteristics of bone ingrowth of tantalum in a transcortical canine model using cylinders implants. They demonstrated that the average extent of bone filling ranged from 63-80% at 52 weeks.

After that, Bobyn *et al.*,^2^ examined the same aspect in a fully functional canine totally hip arthroplasty model. The mean bone ingrowth observed was 16.8% ± 5.7% with higher value (25.1% ± 10.1%) at the peripheral regions where bone-implant contact was most consistent.

Macheras *et al.*,^4^ undertook a radiological study to evaluate the osteoconductive properties of the tantalum bone interface and the possibility to fill an initial gap less than 5 mm. At an average follow-up of seven years, none of the 82 hips showed radiolucencies. Regarding the 25 hips with an initial gap between the outer part of the cup and the bone, none showed signs of loosening and after 24 weeks the gap was filled by bone.

Komarasamy *et al.*,^5^ evaluated 113 consecutive tantalum monoblock cups showing that in the short term the new uncemented tantalum/polyethylene composite acetabular component can yield a satisfactory clinical and radiological outcome and has high patient satisfaction.

We had an opportunity to revise a fixed tantalum cup for recurrent dislocation and study the histology and the bone ingrowth within this new material. To best of our knowledge no similar reports are present in the literature.

The macroscopic observation of the removed cup gave evidence of a difference of the extent of the bone between the periphery and the dome region. This aspect could be explained with two facts. First of all the technique of removal as the dedicated instrument acts as a caliper which has the center inside the liner; thus the diamond blades cut more at the periphery of the cup, leaving more bone attached in the dome region. Secondly, this aspect could be a confirmation of the properties of tantalum in terms of filling minimal gaps which are mostly located in the dome as Macheras said,^4^ probably also as for the quality of the bone in that region.

The bone ingrowth, defined as the percentage of pores filled by bone, is very high, only comparable to the one observed by Bobyn in the simple transcortical canine model (90% vs. 80%). But in a fully functional canine total hip arthroplasty model the same authors reported a much lower value (16.8% ± 5.7%).

Engh *et al.*, in a post-mortem study of porous coated acetabular components observed a mean extent of bone ingrowth of 32%. Pidhorz *et al.*,^6^ in a post-mortem study of titanium fiber-metal porous coated acetabular components observed a mean extent of bone ingrowth of 12.1 ± 8.2%.^7^

Our study which is focused on a single case with a single evaluation and not several ones with a mean value has evident limits, but the difference in bone ingrowth, 90% compared with 10-30% reported in the literature is important and shows the good properties of this new material.^8^,^9^ Regarding the method of measurement, we prefer to use light microscopy and not backscattered electron microscopy^2^,^8^,^9^ due to a better familiarity with the first method and the availability of only one sample already prepared for that microscopy. Despite the different microscopy, the quantitative evaluation was almost the same as we calculated the number of pores filled by bone.

Our feeling is that this new material reaches higher extent of bone ingrowth when compared with other cups.

## References

[1] Bobyn JD, Stckpool G, Hacking SA, Tanzer M, Krygier JJ (1999). Characteristics of bone ingrowth and interface mechanics of a new porous tantalum biomaterial. J Bone Joint Surg Br.

[2] Bobyn JD, Toh KK, Hacking SA, Tanzer M, Krygier JJ (1999). Tissue response to porous tantalum acetabular cups: A canine model. J Arthroplasty.

[3] Gruen TA, Poggie RA, Lewallen DG, Hanssen AD, Lewis RJ, O'Keefe TJ (2005). Radiographic evaluation of a monoblock acetabular component: A multicenter study with 2- to 5-year results. J Arthroplasty.

[4] Macheras GA, Papagelopoulos PJ, Kateros K, Kostakos AT, Baltas D, Karachalios TS (2006). Radiological evaluation of the metalbone interface of a porous tantalum monoblock acetabular component. J Bone Joint Surg Br.

[5] Komarasamy B, Vadivelu R, Bruce A, Kershaw C, Davison J (2006). Clinical and radiological outcome following total hip arthroplasty with an uncemented trabecular metal monoblock acetabular cup. Acta Orthop Belg.

[6] Sporer SM, Paprosky WG (2006). The use of a trabecular metal acetabular component and trabecular metal augment for severe acetabular defects. J Arthroplasty.

[7] Meneghini RM, Lewallen DG, Hanssen AD (2008). Use of porous tantalum metaphyseal cones for severe tibial bone loss during revision total knee replacement. J Bone Joint Surg Am.

[8] Engh CA, Zettl-Schaffer KF, Kukita Y, Sweet D, Jasty M, Bragdon C (1993). Histological and radiographic assessment of well functioning porous-coated acetabular components: A human postmortem retrieval study. J Bone Joint Surg Am.

[9] Pidhorz LE, Urban RM, Jacobs JJ, Sumner DR, Galante JO (1993). A quantitative study of bone and soft tissues in cementless porous-coated acetabular components retrieved at autopsy. J Arthroplasty.

